# COMBINED VENOUS AND ARTERIAL RECONSTRUCTION IN THE TRIANGLE AREA AFTER TOTAL PANCREATODUODENECTOMY

**DOI:** 10.1590/0102-672020210002e1643

**Published:** 2022-06-17

**Authors:** Eduardo de Souza Martins FERNANDES, Jose Maria Assunção MORAES-JUNIOR, Rodrigo Rodrigues VASQUES, Marcos BELOTTO, Orlando Jorge Martins TORRES

**Affiliations:** 1 Department of Gastrointestinal and Transplant Surgery, Hospital São Lucas/Dasa, Rio de Janeiro, Brazil.; 2 Department of Gastrointestinal and Transplant Surgery, Hospital São Domingos/Dasa, São Luiz, Maranhão, Brazil.; 3 Department of Gastrointestinal Surgery, Hospital 9 de Julho/Dasa, São Paulo, Brazil.

**Keywords:** Pancreas, Adenocarcinoma, Pancreaticoduodenectomy, Mesenteric Veins, Mesenteric Arteries, Portal Vein, Hepatic Artery., Pâncreas, Adenocarcinoma, Pancreaticoduodenectomia, Veias Mesentéricas, Artérias Mesentéricas, Veia porta, Artéria Hepática.

A 40-year-old woman presented with ductal adenocarcinoma in the body of the pancreas, involving the celiac trunk (CT) completely (encasement), superior mesenteric artery (SMA) (>180^o^), and superior mesenteric vein (SMV)/portal vein (PV) (>180^o^). After four cycles of neoadjuvant chemotherapy (FOLFIRINOX), she underwent a total pancreatectomy, lymphadenectomy, total mesopancreas excision^1^, and resection of the CT, SMA, and PV/SMV. The stump of the CT was anastomosed to the proper hepatic artery (PHA). A termino-terminal anastomosis was performed in the SMA, and the SMV was anastomosed to the PV. After total mesopancreas excision, the triangle operation is observed (Figure 1a and b) after resection and reconstruction of the three components of the triangle.

**Figure 1 -:**
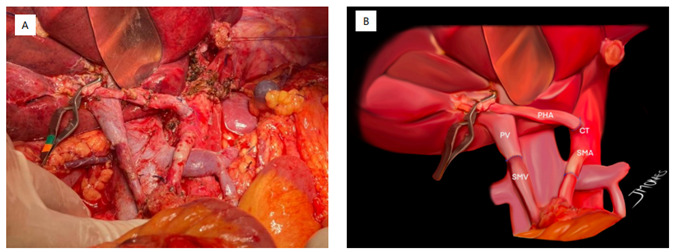
A and B. Triangle operation with resection and reconstruction of the three components. PV, portal vein; SMV, superior mesenteric vein; PHA, proper hepatic artery; CT, celiac trunk; SMA, superior mesenteric artery.

## DISCUSSION

Pancreatoduodenectomy after neoadjuvant chemotherapy is the current treatment in patients with borderline pancreatic ductal adenocarcinoma in the head of the pancreas^1^ ^,^ ^2^ ^,^ ^3^. The total mesopancreas excision concept includes the resection of the lymphatic structures on the right side of the SMA and along the neuronal plexus of the pancreatic head. Complete clearance of this retroperitoneal area may increase the R0 resection rate in patients with adenocarcinoma in the head of the pancreas. This area is an important location of perineural infiltration of tumor cells in patients with pancreatic ductal adenocarcinoma^4^.

Hackert et al^5^ described the term “triangle operation” as a new surgical technique for patients with locally advanced pancreatic ductal adenocarcinoma and stable disease following neoadjuvant therapy. This area is defined by SMV/PV, celiac axis/common hepatic artery, and SMA, representing the typical view after completion of the resection. However, according to the definition of the authors, the procedure should be performed without arterial resection. Recently, Loss et al^6^ and Schneider et al^7^ observed that arterial resection is effective in patients with locally advanced pancreatic cancer after neoadjuvant chemotherapy, with better long-term survival than with palliative treatment. However, this procedure should be performed in experienced pancreatic centers. After neoadjuvant chemotherapy and centers with expertise in pancreatic resection, arterial resection is perfectly possible with acceptable morbidity and mortality.
